# Neurotrophic Effect of Adipose Tissue-Derived Stem Cells on Erectile Function Recovery by Pigment Epithelium-Derived Factor Secretion in a Rat Model of Cavernous Nerve Injury

**DOI:** 10.1155/2016/5161248

**Published:** 2015-12-13

**Authors:** Xin Chen, Qiyun Yang, Tao Zheng, Jun Bian, Xiangzhou Sun, Yanan Shi, Xiaoyan Liang, Guoquan Gao, Guihua Liu, Chunhua Deng

**Affiliations:** ^1^Department of Urology, The First Affiliated Hospital of Sun Yat-sen University, Guangzhou 510080, China; ^2^Department of Urology, The Third Affiliated Hospital of Southern Medical University, Guangzhou 510630, China; ^3^Reproductive Medicine Research Center, The Sixth Affiliated Hospital of Sun Yat-sen University, Guangzhou 510655, China; ^4^Department of Biochemistry, Zhongshan School of Medicine, Sun Yat-sen University, Guangzhou 510080, China

## Abstract

The paracrine effect is the major mechanism of stem cell therapy. However, the details of the effect's mechanism remain unknown. The aim of this study is to investigate whether adipose tissue-derived stem cells (ADSCs) can ameliorate cavernous nerve injury-induced erectile dysfunction (CNIED) rats and to determine its mechanism. Twenty-eight days after intracavernous injection of 5-ethynyl-2-deoxyuridine- (EdU-) labeled ADSCs, the erectile function of all the rats was evaluated by intracavernosal pressure (ICP). The ADSCs steadily secreted detectable pigment epithelium-derived factor (PEDF) *in vitro*. The expression of PEDF increased in the penis of the bilateral cavernous nerve injury (BCNI) group for 14 days and then gradually decreased. On day 28 after the intracavernous injection, the ADSCs group exhibited a significantly increased ICP compared with the phosphate buffered saline- (PBS-) treated group. Moreover, the neuronal nitric oxide synthase (nNOS) and S100 expression in penile dorsal nerves and the smooth muscle content to collagen ratio in penile tissues significantly increased. Furthermore, elevated PEDF, p-Akt, and p-eNOS were identified in the ADSCs group. This study demonstrated that intracavernous injection of ADSCs improved erectile function, repaired the nerve, and corrected penile fibrosis. One potential mechanism is the PEDF secretion of ADSCs and subsequent PI3K/Akt pathway activation.

## 1. Introduction

Prostate cancer (PCa) is the most common cancer in elderly males worldwide. Currently, radical prostatectomy (RP) is the optimal treatment for clinically localized prostate cancer [1]. However, erectile dysfunction (ED) is a frequent complication after RP, which decreases the quality of life of these patients and their partners. The main reason for postprostatectomy ED is cavernous nerve injury and neuropraxia [2]. The subsequent long-term denervation of penile tissue results in cavernous fibrosis and ED [3, 4]. Despite the advances in surgical techniques, such as nerve-sparing and robot-assisted surgery, which may reduce ED incidence, the treatment for postoperative ED remains challenging [5]. Phosphodiesterase type-5 (PDE5) inhibitors, which are the first-line treatment for ED, exhibit a low response in these patients due to apoptosis of corporal smooth muscle and collagen accumulation [6]. Therefore, new treatment strategies for ED after RP are highly desirable.

In the previous decade, exogenous neurotrophic factors such as brain-derived neurotrophic factor (BDNF), vascular endothelial growth factor (VEGF), and glial cell line-derived neurotrophic factor (GDNF) have been investigated in the treatment of peripheral nerve injuries or ED following cavernous nerve injury [7, 8]. However, the short-term and temporary effects of nerve regeneration have limited the direct clinical application of these factors. Furthermore, Boyd and Gordon also demonstrated that high-dose BDNF inhibited motor axonal regeneration, which resulted in the difficulty of BDNF utilization to sustain nerve regeneration in clinical applications [9]. Although the neurotrophic factors delivered by tissue engineering technologies and viral vectors have proven to be effective in cavernous nerve injury-induced erectile dysfunction (CNIED) animal models [10, 11], the biohazard associated with foreign material and virus introduction has hindered their clinical application.

Recently, various stem cells have proven to be effective in the treatment of cavernous nerve injury-induced erectile dysfunction. However, it has been difficult to identify these transplanted stem cells in penile tissues [12–15]. Nevertheless, some studies have shown that bone marrow-derived mesenchymal stem cells (BM-MSCs) secrete several neurotrophic factors such as basic fibroblast growth factor (bFGF), nerve growth factor (NGF), BDNF, VEGF, and insulin-like growth factor 1 (IGF-1). Both BM-MSCs and their conditioned medium were effective in nerve regeneration improvement in diabetic rats [13, 16]. Our previous study also demonstrated that ADSCs could improve erectile function in diabetic erectile dysfunction rats via the secretion of trophic factors such as VEGF, serpin F1 (PEDF), NGF, b-FGF, and IGF-1. The engraftment and differentiation of stem cells were not observed in penile tissues [17, 18]. Like others, we believe that the paracrine effect is the major mechanism for the stem cell therapeutic effect of ED. However, the detailed paracrine mechanisms of ADSCs in the amelioration of cavernous nerve injury-induced erectile dysfunction remain unknown.

PEDF is a 50 kDa glycoprotein that belongs to the noninhibitory and multifunctional serpin group [19]. PEDF was first discovered in the conditioned medium of cultured human fetal retinal pigment epithelial cells and was identified as a neurotrophic factor [20]. The neuroeffect was induced by the activation of nuclear factor *κ*B, which, in turn, induced the expression of neurotrophic factors (NGF, BDNF, and GDNF) and antiapoptotic genes (Bcl2, Bcl-x) in cerebellar granule cells [21]. PEDF is also a potent endogenous inhibitor of angiogenesis [22] that was demonstrated to exert its antitumor activities via antiangiogenesis, tumor cell differentiation, and direct tumor suppression by apoptosis [23]. Interestingly, PEDF can have the opposite effects on endothelial cells of different phenotypes. Although PEDF promotes the apoptosis of endothelial cells, it exerts a synergistic proliferative effect on endothelial cells with VEGF [24]. Current studies shows that PEDF is widely expressed throughout fetal and adult tissues and has multiple important functions including differentiation, neuroprotection, and antiangiogenic effects on different cells or tissues [25]. Recently, Vigneswara et al. demonstrated that PEDF has neuroprotective and axogenic effects on retinal ganglion cells in an optic nerve crush injury model [26]. Therefore, in the present study, we analyzed PEDF secretion in the ADSCs-treated group to explore the mechanism of ADSCs therapy in a rat model of cavernous nerve injury-induced erectile dysfunction.

## 2. Materials and Methods

### 2.1. Ethics Statement

Eighty-eight male Sprague-Dawley (SD) rats were purchased from the Animal Center of Sun Yat-Sen University (Guangzhou, China) and used in this study. The animals were housed in a standard specific pathogen-free environment with free access to laboratory chow and water. The rats were monitored at least once a day. Eight rats (60–80 g) for ADSC primary culture were sacrificed using diluted, buffered pentobarbital sodium (30 mg/kg) solution via an intraperitoneal (IP) injection followed by the cervical dislocation method. Eighty rats (250–300 g) were first anesthetized with pentobarbital sodium (40 mg/kg) via an IP injection. After the experiments, they were sacrificed with an overdose of pentobarbital sodium (200 mg/kg) anesthesia via an IP injection. The experiments were approved by the Institutional Animal Care and Use Committee of Sun Yat-Sen University.

### 2.2. Study Design

Eighty SD rats (250–300 g, 8–10 weeks old) were divided into 4 groups: (a) sham, (b) bilateral cavernous nerve injury (BCNI), (c) phosphate buffered saline (PBS), and (d) ADSC groups. The sham group underwent laparotomy only (*n* = 32). The remaining rats (*n* = 48) underwent a bilateral cavernous nerve crush injury and were then randomly divided into three groups: the BCNI group (*n* = 32), the intracavernous injection with ADSCs (ADSC group, *n* = 8), or the PBS (PBS group, *n* = 8). A total of four rats died in the sham and BCNI groups and were excluded in this study. On days 2, 7, and 14, the sham and BCNI groups (*n* = 8 per group) were randomly sacrificed, and the penile tissues were harvested for Western blotting analyses. Four weeks after treatment, the erectile function of the four groups (*n* = 8, resp.) was evaluated using the intracavernosal pressure (ICP) and ICP/mean arterial pressure (MAP) ratio before the penile tissues were harvested.

### 2.3. Isolation, Culture, Characterization, and Labeling of ADSCs

Eight SD rats (60–80 g, 3 weeks old) were sacrificed, and adipose tissues were isolated from the bilateral groin. The ADSCs were cultured according to our previous protocol [27]. Briefly, the tissue was incubated in 0.1% collagenase Type I A (Sigma-Aldrich, St. Louis, MO, USA) for 50 minutes at 37°C and shaken for 30 seconds at 15-minute intervals. After filtration with a 100 *μ*m cell strainer and centrifugation, the adipose tissue stromal-vascular fraction (SVF) was immersed in red blood cell lysis buffer for 5 minutes at 4°C. After the remaining cells were centrifuged and rinsed with PBS, they were resuspended in Dulbecco's Modified Eagle's Medium: Nutrient Mixture F-12 (DMEM/F12) supplemented with 10% fetal bovine serum (FBS, Gibco, Life Technologies, USA) and then cultured in 25 cm^2^ cell culture flasks. The cells were passaged at approximately 80% confluence. The rat ADSCs were identified according to our previously described methods [17]. Fluorescence-conjugated antibodies (Table 1) were incubated with ADSCs at 4°C for 30 minutes. Cell surface antigens of ADSCs (passage 3) were determined by flow cytometry analysis (Calibur BD Biosciences, Franklin Lakes, NJ). Osteogenic and adipogenic differentiation of ADSCs was assessed by Alizarin Red S or Oil Red O staining, respectively.

For the labeling of ADSCs, culture medium for ADSCs was renewed at 48 h before cell transplantation, and 5-ethynyl-2-deoxyuridine (EdU) at a final concentration of 10 *μ*M was added according to the manufacturer's instructions (Click-iT EdU Imaging Kits, Invitrogen, Carlsbad, CA, USA).

### 2.4. PEDF Secreted by Cultured Rat ADSCs

In total, 2 × 10^5^ ADSCs at passages 2, 3, 4, 5, 8, and 10 were seeded in 6-well plates and incubated with serum-free DMED/F12 under standard conditions (5% CO_2_, 37°C) for 24 hours. The culture supernatant from the ADSCs was subsequently collected and analyzed using a rat PEDF ELISA kit (CUSABIO, Wuhan, Hubei province, China) according to the manufacturer's instructions. Serum-free medium served as the control. Briefly, 100 *μ*L of standard or samples were incubated with precoated 96-well plates for two hours on a rocking platform at 37°C, followed by incubation with biotin antibody and HRP for 1 hour at 37°C. The OD values were read at the 450 nm wavelength using a microplate reader (Model 680, BIO-RAD, USA).

### 2.5. A Rat Model of Erectile Dysfunction Induced by Bilateral Cavernous Nerve Crush Injury and* In Vivo* Implantation

A rat model of cavernous nerve injury-induced erectile dysfunction was established according to our previously published methods [28]. Briefly, adult male SD rats were anesthetized with pentobarbital sodium (40 mg/kg) via an intraperitoneal injection. The animals were subsequently fixed in a supine position on a thermally regulated surgical table. After the surgical area was shaved and iodinated for sterilization, a lower midline abdominal incision was performed and the prostate glands were exposed. For the sham group, the abdomen was then closed. The major pelvic ganglion (MPG) and cavernous nerve were identified posterolaterally on either side of the prostate under a dissecting microscope (10x magnification). BCNI was induced via direct perturbation of the nerve 5 mm distal to the MPG with mosquito hemostatic forceps (J31020, Jinzhong, Shanghai, China) for 1 minute. After the penis was exposed, a 1 × 10^6^ EdU-labeled allogeneic rat ADSCs (passage 3) suspension in 0.2 mL PBS or 0.2 mL PBS alone was injected into both corpora cavernosa at the middle level. Prior to the injection, an elastic band was applied at the base of the penis and maintained for 2 min. No animals were given any immunosuppressant before or after surgery.

### 2.6. Erectile Function Assessment

Erectile function was evaluated using the mean arterial pressure (MAP) and intracavernosal pressure (ICP) four weeks after intracavernous injection as previously described by our group [18, 29]. Briefly, 8 rats per group were anesthetized with pentobarbital sodium (40 mg/kg) via an intraperitoneal injection. The left carotid artery was subsequently cannulated with a PE-50 catheter filled with 250 IU/mL heparin solution to measure the MAP. A 25 G needle was punctured into one side of the penile crus and connected to another pressure transducer for the ICP measurement. With a midline laparotomy, the cavernosal nerve was identified and isolated. A bipolar hook electrode attached to a signal generator (BL-420F, Taimeng, Chengdu, China) was placed around the left CN for stimulation. Monophasic rectangular pulses (stimulus parameter settings of 0.2 ms width, 1.5 mA, and frequency at 20 Hz, and duration of 60 s) were recorded and analyzed by computer with BL New Century 2.0 software (Taimeng, Chengdu, China). Erectile function was assessed as the ratio of ICP (mmHg)/MAP (mmHg). The penis was then harvested for Western blotting and histological analyses.

### 2.7. Western Blotting

The penis protein levels were analyzed by Western blotting on the Odyssey infrared imaging system (LI-COR, Nebraska, USA). In brief, the protein samples of six rats per group were separated by 6–12% sodium dodecyl sulfate polyacrylamide gel (10–20 *μ*g per lane). Primary antibodies were applied after the proteins were transferred to nitrocellulose membranes; GAPDH was used as the loading control. Signals were obtained in the linear range of detection and quantified with the Odyssey infrared imaging system. The data were analyzed and presented as the relative density of each protein relative to GAPDH. The primary antibodies were rabbit anti-PEDF polyclonal antibody (dilution 1 : 1000, Santa Cruz Biotechnologies, Santa Cruz, CA, USA, sc-25594), rabbit anti-phospho-eNOS polyclonal antibody (dilution 1 : 500, Abcam, Cambridge, UK, ab75639), rabbit anti-phospho-Akt monoclonal antibody (dilution 1 : 1000, cell signaling technology, Danvers, MA, USA, #4060), and mouse anti-GAPDH monoclonal antibody (dilution 1 : 5000, Proteintech, USA, 60004-1-Ig.).

### 2.8. Histological Analysis

The fresh tissues were fixed in cold 2% formaldehyde followed by overnight immersion in a buffer solution containing 30% sucrose. Then, the tissues were embedded in optimum cutting temperature (O.C.T.) compound (Sakura Finetek, Torrance, CA, USA) and stored at −80°C. For immunohistochemical staining, the sections were cut at 6 *μ*m and adhered to charged slides. After rehydration with PBS, the tissue sections were rinsed with hydrogen peroxide and methanol to block endogenous peroxidase activity. The sections were washed 3 times in PBS and incubated with 3% goat serum/PBS/0.3% triton X-100 (Sigma-Aldrich, St. Louis, MO, USA) for 1 hour; the tissues were subsequently incubated overnight at 4°C with mouse anti-nNOS monoclonal antibody (dilution 1 : 200, Santa Cruz Biotechnologies, Santa Cruz, CA, USA, sc-5302). The slides were washed with PBS and immunostained using the Polink-1 HRP DAB Detection System (PV-6002, GBI, USA). Then, the sections were stained with DAB followed by hematoxylin counterstaining.

For immunofluorescence staining, the penile tissues embedded in O.C.T. were processed at 10 *μ*m and incubated overnight at 4°C with rabbit anti-S100 antibody (dilution 1 : 500, Abcam, Cambridge, UK, ab868). Thereafter, the sections were immersed in a 1 : 500 dilution of secondary antibody conjugated with Alexa-488 Fluor (Invitrogen, Carlsbad, CA, USA) for 1 hour at room temperature. To visualize EdU-labeled ADSCs and to determine whether they could differentiate into Schwann cells, the slides were incubated with Click-iT reaction cocktail (Invitrogen, Carlsbad, CA, USA) according to Lin's procedure with minor modifications [30]. Briefly, the sections were rinsed with PBS 3 times. Then, they were incubated with freshly prepared Click-iT reaction cocktail containing Alexa-594 Fluor for 30 minutes at room temperature. Nuclear staining was performed with 4′,6-diamidino-2-phenylindole (DAPI; Invitrogen, Carlsbad, CA, USA).

For Masson's trichrome staining, the tissues were fixed in 4% paraformaldehyde, followed by ethanol gradient dehydration and fixation in paraffin. The sections were cut at 5 *μ*m and stained according to Masson's trichrome-staining protocol for connective tissue and smooth muscle.

### 2.9. Quantitative Image Analysis

Computerized histomorphometric analysis was performed using Image-Pro Plus 6.0 software (Media Cybernetics, Bethesda, MD, USA). Specifically, 6 rats per group and three penile tissue sections per rat were chosen for statistical analysis. The sections were photographed and recorded with a digital camera coupled to a Leica microscope. The ratio of the nNOS- and S100-positive fibers over the total area of the nerve in pixels was calculated at a magnification of ×400, which included all branches of the penile dorsal nerves per section. For the analysis of Masson's trichrome staining, three sections per animal and 6 rats per group were analyzed using Image-Pro Plus 6.0 software. Magnification images (100x) were analyzed for smooth muscle (stained in red) and collagen (blue) in pixels and expressed as the ratio of smooth muscle/collagen.

### 2.10. Statistical Analysis

Overall comparisons between the groups were analyzed using Graph Pad Prism v.6.0 software (Graph Pad Software, La Jolla, CA, USA). The continuous variables were expressed as the mean ± SD. Multiple groups were compared using one-way ANOVAs followed by a Student-Newman-Keuls post hoc test. *P* < 0.05 was considered statistically significant.

## 3. Results

### 3.1. Characterization of the Rat ADSCs

**Figure 1:**
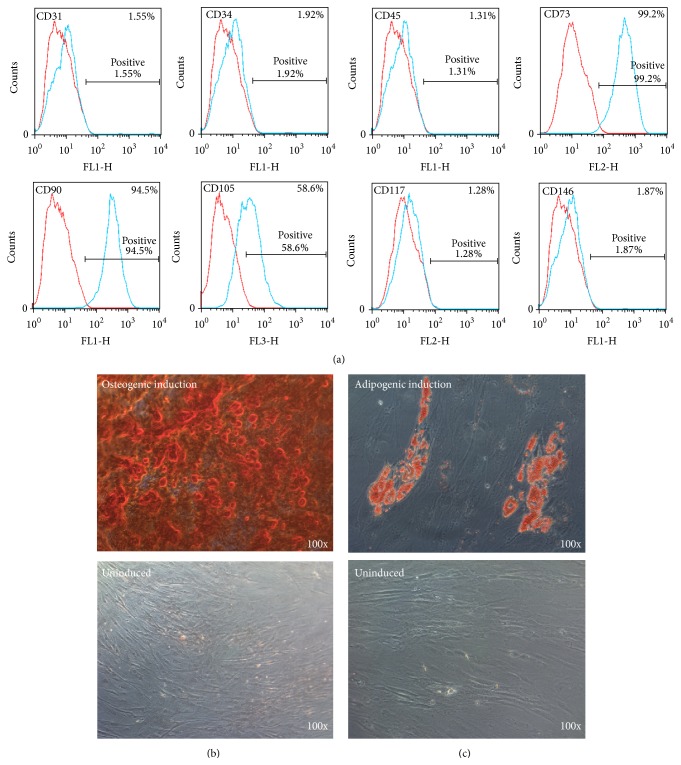
Characterization of the rat ADSCs. (a) FACS analysis revealed that surface antigen expression of ADSCs (*P3*) was consistent with that of mesenchymal stem cells. Representative images of (b) osteogenic-induced ADSCs with Alizarin red S staining and (c) adipogenic-induced ADSCs with Oil Red O staining. Original magnification is ×100; ADSCs = adipose tissue-derived stem cells; FACS = fluorescence activated cell sorting.

The characterization of ADSCs used in this study was identified in the same manner as in our previous published study. These rat ADSCs expressed CD73, CD90, and CD105 but did not express CD146. In addition, they did not express the hematopoietic and endothelial lineage markers CD31, CD34, CD45, and CD117. In addition, osteogenic and adipogenic inducing differentiation of ADSCs further demonstrated the multipotent differentiation capacity of rat ADSCs (Figure 1).

### 3.2. PEDF Secreted by Rat ADSCs* In Vitro*

**Figure 2:**
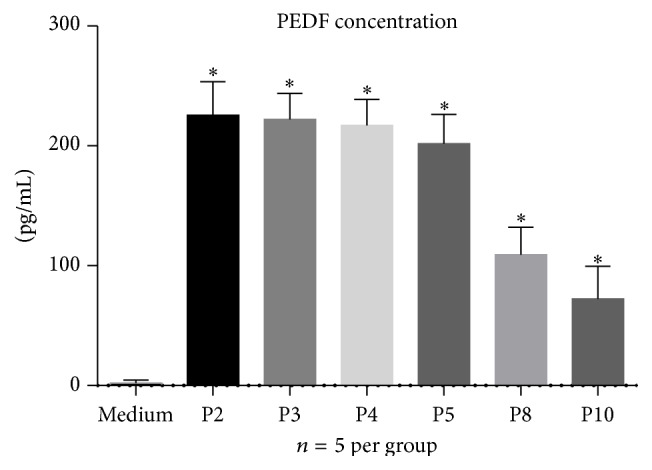
PEDF in different passages of rat ADSC-conditioned medium. Five samples per passage were analyzed using a rat PEDF ELISA kit. The different passages of the rat ADSCs secreted a detectable level of PEDF in cultured serum-free DMEM/F12 medium. ^*∗*^ *P* < 0.05 compared with the DMEM/F12 medium group only. PEDF = pigment epithelium-derived factor; ADSCs = adipose tissue-derived stem cells; DMEM/F12 = Dulbecco's Modified Eagle's Medium/F12.

ADSCs could secrete detectable levels of PEDF as demonstrated by ELISA* in vitro*. The PEDF concentrations of P2, P3, P4, and P5 in the ADSC supernatants were 225.6 ± 12.48, 222.0 ± 9.82, 217.2 ± 9.64, and 201.8 ± 10.96 pg/mL, respectively. In general, the PEDF released by ADSCs before passage 5 was greater than 200 pg/mL and was subsequently decreased (Figure 2).

### 3.3. Erectile Function Measurements

**Figure 3:**
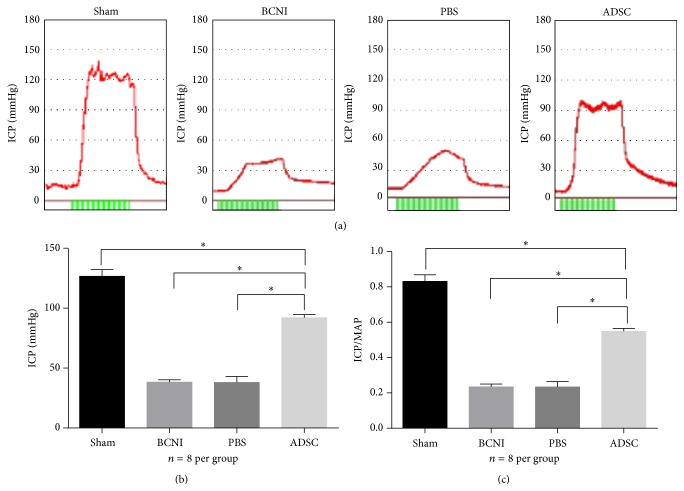
ADSCs intracavernous injection improved erectile function in the BCNI rat model. (a) Representative ICP tracing responses of each group (*n* = 8 per group) to the stimulation of the cavernous nerve 28 days following the intracavernous injection of ADSCs or PBS. Green bar represents an electrical stimulus duration of 60 seconds. (b) The effects of ADSCs treatment on the ICP increase. ^*∗*^ *P* < 0.05. (c) The ratio of ICP to MAP was calculated for each group. ^*∗*^ *P* < 0.05. BCNI = bilateral cavernous nerve injury; ICP = intracavernosal pressure; MAP = mean arterial pressure.

To determine erectile function, the intracavernosal pressure of 8 rats per group was assessed at 4 weeks after ADSCs intracavernous injection. Representative ICPs of all 4 groups were recorded using the tracing response to the stimulation of the cavernous nerve. The sham group exhibited significantly increased ICP and ICP/MAP ratio compared with the bilateral cavernous nerve injury or PBS-treated groups. Furthermore, the ADSCs-treated group exhibited a significantly increased ICP/MAP ratio of the cavernous nerve injury-induced erectile dysfunction rats compared with the PBS-treated group. The average ICP and ICP/MAP ratio of the ADSCs-treated group were 92.06 ± 0.94 mmHg and 0.5488 ± 0.0058, respectively, which represented 72.66% and 66.01% of that of the sham group, respectively (Figure 3).

### 3.4. Western Blotting Analysis

**Figure 4:**
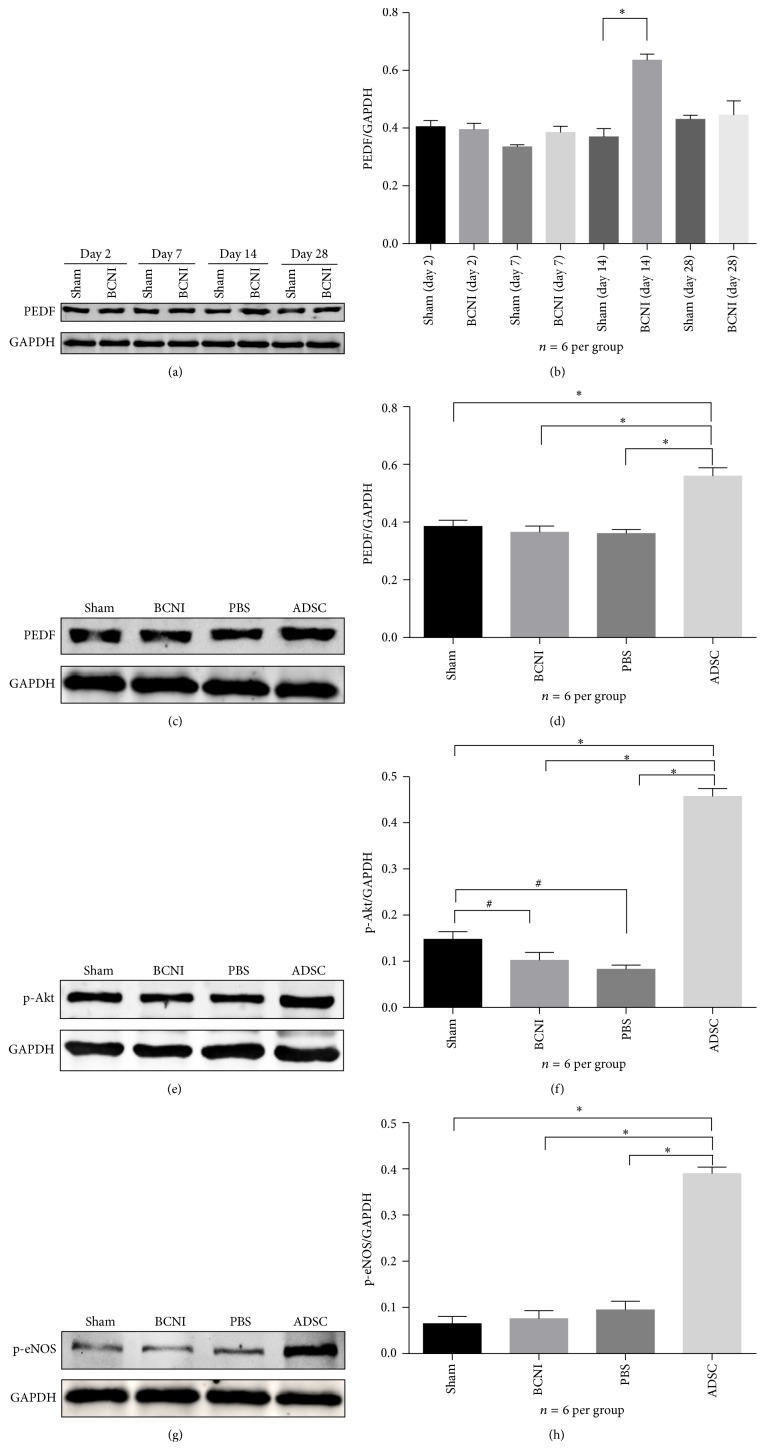
Western blotting analyses of PEDF, p-Akt, and p-eNOS protein expression in the rat penile tissues of all groups. (a) Representative Western blot and (b) quantitative analysis identified increased PEDF expression in the bilateral cavernous nerve injury (BCNI) group compared with the sham group at day 14, which recovered to the background level at day 28. ^*∗*^ *P* < 0.05. (c)-(d) Increased PEDF expression in the ADSCs treatment group compared with the other groups at day 28. ^*∗*^ *P* < 0.05. (e)-(f) Increased p-Akt expression in the ADSCs group compared with the other groups at day 28 and increased p-Akt expression in the sham group compared with the bilateral cavernous nerve injury and PBS groups. ^*∗*^ *P* < 0.05 and ^#^ *P* < 0.05. (g-h) Increased p-eNOS expression in the ADSCs treatment group compared with the other groups at day 28. ^*∗*^ *P* < 0.05. The data were normalized to GAPDH protein expression. *n* = 6 per group. p-Akt = phosphorylated Akt; p-eNOS = phosphorylated endothelial nitric oxide synthase; GAPDH = glyceraldehyde 3-phosphate dehydrogenase.

The expression of PEDF, p-Akt, and p-eNOS in penile tissue extracts of 6 rats per group was analyzed by Western blotting. Densitometry indicated that the PEDF level of the BCNI group was significantly increased compared with the sham group at day 14 and declined rapidly to the baseline level by day 28 (Figures 4(a) and 4(b)). However, the expression of PEDF in the ADSC group at day 28 was significantly increased compared with those of the other groups (Figures 4(c) and 4(d)). Furthermore, the p-Akt and p-eNOS proteins, two dominant factors of the PI3K/Akt pathway, were also increased in the ADSC group compared with those of all other groups (Figures 4(e)–4(h)).

### 3.5. Distribution of EdU-Positive ADSCs in Penile Tissues

**Figure 5:**
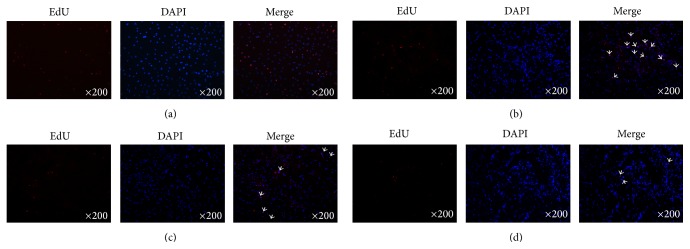
EdU labeling of ADSCs and tracking of EdU-labeled ADSCs in the penile tissues after cell transplantation. (a) Representative image of ADSCs labeled with EdU at 10 *μ*M and stained with Alexa Fluor 594 (red fluorescence) and Hoechst 33342 (blue fluorescence). Original magnification is 200x. (b)–(d) Penis tissues were harvested at 7 d, 14 d, and 28 d after ADSCs transplantation and stained with Alexa Fluor 594 (red fluorescence) and DAPI (blue fluorescence). The merged image showed that the EdU and DAPI double positive cells displayed a time-dependent decreasing pattern in the penis tissue. Original magnification is 200x. EdU = 5-ethynyl-2-deoxyuridine; DAPI = 4′,6-diamidino-2-phenylindole.

Incubation of ADSCs in EdU containing media showed bright red fluorescence in the nuclei when stained with Alexa-594* in vitro*. The detection of EdU colocalized with Hoechst 33342, which was the blue fluorescent nuclear label. However, only 30% of Hoechst 33342-positive nuclei were EdU-positive (Figure 5(a)). EdU-labeled ADSCs were also examined at days 7, 14, and 28 after intracavernous injection. The results demonstrated that no more than 50 ADSCs per field (200x magnification) were detected at each time point. Distribution of EdU-positive ADSCs was mainly in the penile cavernous sinus. Moreover, few ADSCs could be found in penis tissue 28 days after injection. The EdU-positive ADSCs in the penis displayed a time-dependent decreasing pattern by intracavernous injection (Figures 5(b)–5(d)).

### 3.6. Histomorphometric Analysis of Penile Tissue

**Figure 6:**
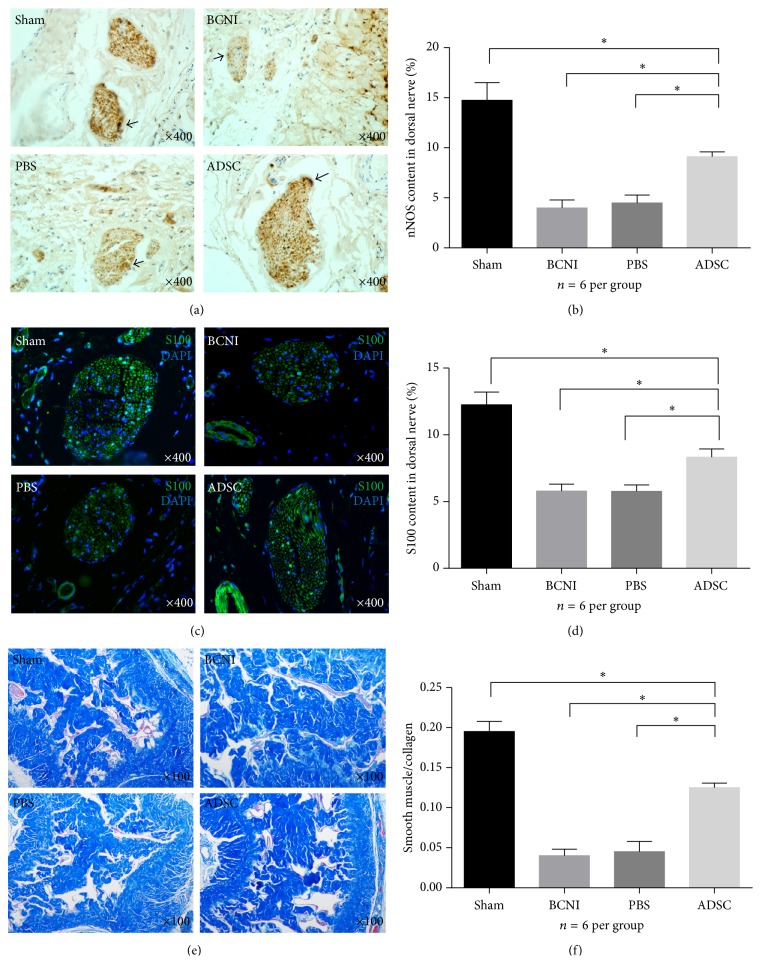
*Histomorphometric Analysis of Penile Tissue.* (a) Representative images are presented for the nNOS-positive fibers (black arrow indicated) in the penile dorsal nerves of each experimental group via immunohistochemical analysis. Original magnification is ×400. *n* = 6 per group. (b) Quantitative analysis of the nNOS-positive fibers to the total area of penile dorsal nerves in pixels calculated by Image-Pro Plus 6.0 software at a magnification of ×400. ^*∗*^ *P* < 0.05. (c) Representative images of the S100-positive fibers in the penile dorsal nerves of each experimental group via immunofluorescence analysis. Original magnification is ×400. *n* = 6 per group. (d) Quantitative analysis of the S100-positive fibers to the total area of penile dorsal nerves in pixels calculated by Image-Pro Plus 6.0 software at a magnification of ×400. ^*∗*^ *P* < 0.05. (e) Representative images of the penile tissue stained with Masson's trichrome in all experimental groups. Smooth muscle is stained red, and collagen and connective tissue are stained blue. Original magnification is ×100, *n* = 6 per group. (f) The ratio of smooth muscle to collagen and connective tissue area in the corpus cavernosum was calculated in each group. ^*∗*^ *P* < 0.05. nNOS = neuronal nitric oxide synthase.

The nNOS and S100 expression in the penile dorsal nerves of each group (6 rats per group and 3 sections per rat) were determined by immunohistochemistry or immunofluorescence. The ratio of nNOS-positive fibers to penile dorsal nerve areas in the sham, bilateral cavernous nerve injury, PBS, and ADSC groups was 14.74%, 3.99%, 4.49%, and 9.11%, respectively. Compared with the sham group, the nNOS-positive fibers in the dorsal penile nerves were significantly decreased in the bilateral cavernous nerve injury and PBS groups. Intracavernous injection of ADSCs improved the number of nNOS-positive fibers compared with the bilateral cavernous nerve injury and PBS-treated groups. However, the nNOS expression level in the ADSCs-treated group was still lower than that of the sham group (Figures 6(a)-6(b)). The ratio of S100-positive fibers to the dorsal penile nerve areas in the sham, bilateral cavernous nerve injury, PBS, and ADSCs groups was 12.26%, 5.79%, 5.77%, and 8.34%, respectively. S100 and nNOS expression in all four groups showed a similar trend in penile dorsal nerves (Figures 6(c)-6(d)). Masson's trichrome staining indicated the development of corpus cavernosum fibrosis in the bilateral cavernous nerve injury group. ADSCs treatment significantly increased the smooth muscle content and ameliorated fibrosis in the corpus cavernosum. The ratios of smooth muscle/collagen in the sham, bilateral cavernous nerve injury, PBS, and ADSCs groups were 0.195 ± 0.013, 0.040 ± 0.008, 0.045 ± 0.013, and 0.125 ± 0.006, respectively. This ratio in the bilateral cavernous nerve injury and PBS groups was significantly decreased compared with that of the sham group. The ADSCs group partially improved smooth muscle content and decreased penile fibrosis compared with that of the bilateral cavernous nerve injury or PBS groups; however, the ratio of smooth muscle/collagen was still lower than that of the sham group (Figures 6(e)-6(f)).

## 4. Discussion

In the present study, we systematically demonstrated the typical expression of PEDF in a cavernous nerve injury-induced erectile dysfunction rat model. Furthermore, ADSCs injected into cavernous nerve injury-induced erectile dysfunction rat cavernous tissues could ameliorate erectile dysfunction and penile fibrosis. One potential mechanism was the neurotrophic effect of ADSCs via PEDF secretion, which may thereby activate the PI3K/Akt pathway.

Recently, Calenda demonstrated the early mobilization of PEDF (*SERPINF1*) involved in nerve repair and neuroprotection via the analysis of a whole genome microarray in the rat MPG after cavernous nerve injury. The PEDF gene was upregulated (3-fold compared with the control) at 48 hours after cavernous nerve injury but recovered to the normal level after 14 days [31]. However, this effect was only demonstrated at the gene level and was not found for protein expression. Therefore, our study further verified the increased PEDF protein expression at day 14, which recovered to the baseline level by day 28 after bilateral cavernous nerve injury. These findings indicated the temporary and insufficient PEDF protein expression in a rat model of cavernous nerve injury-induced erectile dysfunction.

Erectile dysfunction typically developed at 18–24 months after patients underwent nerve-sparing RP [32]. Although a majority of axons remained intact, Wallerian degeneration was initiated rapidly after nerve injury, and the inflammatory reaction could cause axon damage [33]. Chronic denervation of the erectile tissues caused penile hypoxia, fibrosis, and eventually ED in rats [3]. Therefore, early interventions, such as application of stem cells or neurotrophic factors, are highly desirable for the repair of the damaged cavernous nerve. Fandel et al. demonstrated that cavernous nerve injury attracted intracavernously injected ADSCs to the MPG by increasing stromal cell-derived factor-1 (SDF-1) expression. ADSCs exerted their neuroregenerative effects on the cell bodies of injured nerves, resulting in the enhancement of erectile function [34]. In our study, ADSCs could steadily secrete PEDF for at least five passages* in vitro*. Furthermore, PEDF expression in penile tissues was increased in the ADSCs-treated group at day 28 compared with that of the control groups* in vivo*, which indicated that ADSCs could increase PEDF protein expression for at least 4 weeks.

Crawford et al. reported that Schwann cells could produce PEDF, which in turn enhanced the survival and growth of Schwann cells [35]. Some researchers also discovered that ADSCs could differentiate into Schwann cells under specific conditions* in vitro* [36, 37]. However, in a series of preclinical studies regarding the cavernous nerve injury-induced erectile dysfunction treatment, only a small number of intracavernously injected labeled mesenchymal stem cells (MSCs) could be detected in the penile tissues even though the erectile function and penile structure of cavernous nerve injury-induced erectile dysfunction rats were improved significantly. A shared conclusion was that paracrine effects from MSCs are exerted on damaged cavernous nerves [12, 13, 34]. Our present study suggested a similar result. Intracavernously injected labeled ADSCs demonstrated a time-dependent decreased pattern in the penis. Only a few EdU-positive cells could be tracked 28 days after cell transplantation, most of which were localized in the cavernous sinus. Scarce EdU-labeled ADSCs were observed in penile dorsal nerves expressing the Schwann cell marker S100. However, we found that the expression of S100 in penile dorsal nerves was significantly higher in the ADSCs-treated group than in the bilateral cavernous nerve injury or PBS-treated groups. This demonstrated that ADSCs could partially recover the impaired Schwann cells. Considering the absence of evidence on ADSCs engraftment and differentiation, we speculate that the therapeutic effect was due to the paracrine mechanism of ADSCs. Furthermore, increased PEDF expression, increased nNOS-positive fiber numbers, and decreased penile fibrosis were identified in the ADSCs-treated group, which indicated that ADSCs could recover the impaired never function in CNIED rats via the neurotrophic and neuroprotective effects of PEDF.

Accumulating evidence has verified the therapeutic effects of ADSCs for the treatment of the CNIED rat model [12, 34]. Although the paracrine mechanism of stem cell therapy for ED has gradually become accepted, it has not been fully understood [38]. Sanchez et al. demonstrated that the PEDF protective effect on oxidant injured cortical neurons* in vitro* was blocked by an inhibitor of PI3K or ERK [39]. More importantly, PI3K/Akt dependent phosphorylation and further activation of eNOS mediated a sustained penile erection [40]. In our study, we demonstrated increased expression of phosphorylated Akt (p-Akt) and phosphorylated eNOS (p-eNOS) in the ADSC-treated group compared with those of the other groups. Taken together, evidence suggests that it is reasonable to conclude that one potential mechanism for ADSCs in the correction of CNIED occurs via the secretion of PEDF and subsequent PI3K/Akt pathway activation.

However, the present study does have several limitations. First, although the PEDF secretion of ADSCs was tested* in vitro*, the different microenvironment* in vivo* may change the secretion pattern of ADSCs. Second, our current proof regarding the neurotrophic effect of ADSCs via the PI3K/Akt pathway is limited. Therefore, our further studies aim to explore the effect of PEDF on explant cultures of MPG with attached cavernous nerve fragments* in vitro*. ADSCs overexpressing and lacking PEDF will be further investigated in the treatment of rat cavernous nerve injury-induced erectile dysfunction model.

## 5. Conclusions

In summary, PEDF may be involved in nerve regeneration and neuroprotection in a rat model of cavernous nerve injury-induced erectile dysfunction. However, the neurotrophic effect of PEDF is temporary and insufficient. Intracavernous injection of ADSCs improves erectile function, repairs the nerve, and ameliorates penile fibrosis. One potential mechanism of ADSC treatment in cavernous nerve injury-induced erectile dysfunction is the nerve regeneration induced by PEDF secretion and subsequent PI3K/Akt pathway activation.

## Figures and Tables

**Table 1:** Details of the fluorescence-conjugated antibodies used in flow cytometry analysis of ADSCs.

Primary antibody	Company/catalog #	Antibody dilution		
		WB	IHC	FACS
CD31-FITC	Abdserotec/MCA1334FA			1 : 10
CD34-FITC	Santa Cruz/sc-7324 FITC			1 : 10
CD45-FITC	BD/561867			1 : 5
CD73-PE	BD/550741			1 : 5
CD90-FITC	Biolegend/202503			1 : 5
CD105-PE/Cy7	Biolegend/120413			1 : 10
CD117-PE	Biolegend/105807			1 : 10
CD146-FITC	Biolegend/134705			1 : 10
